# Harnessing artificial intelligence for mental health care

**DOI:** 10.1016/j.ebiom.2025.105563

**Published:** 2025-01-14

**Authors:** eBioMedicine

According to WHO, mental disorders will become the leading cause of the global burden of disease by 2030. A cross-national analysis of population surveys from 29 countries published in *The Lancet Psychiatry* in 2023 estimated that half of the world population would have one or more mental disorders by age 75 years. Delivering effective solutions to meet increasing demand is crucial for accurate diagnosis and precision treatment of these disorders. The integration of artificial intelligence (AI) could transform mental health care by potentially improving early detection and personalised treatment, making services more efficient and accessible.

Machine-learning algorithms, deep-learning models, and large-language models (LLMs) have been explored as tools to assist with early diagnosis, subtype identification, and personalised treatment of mental disorders. Typical examples of AI predictive models are those based on medical images such as x-ray, MRI, or CT. A large, international, multicentre study published in *Molecular Psychiatry* in 2024 developed a classifier based on structural MRI data to predict psychosis onset in adolescent individuals. The classifier could distinguish between individuals who later developed a psychotic disorder and healthy control individuals with 85% accuracy in the training set and 68% in the test set. Performance was 73% accuracy in the independent validation dataset, indicating its real-world applicability for early detection of psychotic disorders during adolescence.

Traditional diagnosis of mental disorders often relies on self-report or clinical observations regarding the number and types of symptoms of distress or impairment. Although convenient for the identification of mental disorders, traditional diagnosis is often subjective and does not reflect the interaction between genetic factors and environmental influences. In their paper published in *Cell* in 2024, Liu and colleagues analysed a large dataset of more than 11,000 US adolescents from the Adolescent Brain Cognitive Development project, which contained clinical diagnostic data, wearable device records, and genomic information. Using machine-learning algorithms, the authors constructed digital phenotypes that could accurately identify psychiatric disorders. These digital phenotypes not only improved the diagnostic accuracy of psychiatric diseases, but also showed the ability to reveal multiple genetic loci associated with behavioural and psychiatric disorders in genome-wide association studies, increasing understanding of the disorders. By linking behaviour, genetics, and wearable data generation, this study allowed us to see application scenarios of AI in research, diagnosis, and treatment of mental disorders and other diseases.

To further understand the biological and clinical heterogeneity of psychiatric disorders, Zhen and colleagues (*NeuroImage*, 2024) analysed functional MRI by prototype learning and developed a novel graph neural network architecture, known as the BPI-GNN, for the diagnosis and subtyping of mental disorders. The BPI-GNN could effectively distinguish between people with autism spectrum disorder, major depressive disorder, or schizophrenia and healthy control individuals and could identify subtype-specific brain network connections. This work displays potential to address existing concerns of poor reproducibility and interpretability in using graph neural networks for diagnoses.

With the rapid digital transformation process of health-care facilities, electronic health records (EHRs) maintained by health-care providers can also be used for patient management. A study published in 2024 by *The Lancet Digital Health* reported a transformer-based pipeline that could convert unstructured data from EHR documents, such as doctor's notes and imaging reports, into structured, coded concepts to generate a multiyear timeframe about a patient's future risk of disorders, provide differential diagnosis, and offer recommendations on medicines to be used. This model was validated in multiple datasets covering both mental illness and physical diseases from different hospitals in Europe and the USA. Although the model cannot be used for clinical decision support in its current form, the authors have made it publicly available (https://foresight.sites.er.kcl.ac.uk).

The complexity of the diagnosis of mental disorders is reflected in the interaction between genetic factors, environmental influences, and the high heterogeneity of individual behaviour and psychological characteristics. As a result, although countless AI models have been developed for diagnosis or prognosis of mental disorders, most cannot be applied in clinical practice due to poor performance. A study published in *Science* in 2024 tested the performance of machine-learning prediction models with randomised controlled trial datasets that assessed the efficacy of antipsychotic medications for the treatment of schizophrenia. Machine-learning models could achieve excellent performance in one dataset, even when the dataset was a large, international, multicentre clinical trial. However, the performance became uncertain when that same model was tested in real-world, independent clinical trials, suggesting that predictive models based on specific datasets should be carefully applied in future studies unless the heterogeneity of patients is minimised and recruiting procedures, such as inclusion criteria and treatment protocols, are highly consistent.

AI-driven tools also have the potential to improve the accessibility of mental health therapy by helping therapeutic exercises or providing supportive therapy sessions. For example, advancements in LLMs have enhanced the therapeutic potential of virtual reality. In a paper published in *NPJ Digital Medicine* in 2024, Spiegel and colleagues developed an extended-reality AI assistant that merged virtual reality and AI for precision mental health assistance based on cognitive behavioural therapy. After recording conversations with people with mild-to-moderate depression or anxiety, the assistant transcribed the speech into text, responded via Generative Pre-trained Transformer 4, and played the output to the person together with a sentiment analysis of meta-data controlling expressions. Although the assistant still needs to be optimised for a deeper engagement with users, it has potential advantages over traditional therapy for people who feel uncomfortable talking to therapists and provides a supplementary approach to meet the increasing need of psychological intervention. Another observational study published in *Nature Medicine* in 2024 suggested that AI could reduce the accessibility gap for people of minoritised ethnicity in the UK. The authors assessed the influence of a personalised, AI-enabled chatbot on self-referral efficiency in the National Health Service, and found that implementation of the self-referral chatbot promoted more referrals by people of minoritised ethnicity compared with control services. Feedback provided by 42,332 individuals who completed the self-referral through the chatbot suggested that the chatbot could have removed potential barriers that people of minoritised ethnicity usually experience when seeking mental health treatment, suggesting that AI could contribute to overcoming the pervasive inequality in mental health care.

The application of AI in mental health care is not without challenges. One major concern is data security and the protection of sensitive patient information. Moreover, maintaining an optimal balance between AI involvement and human oversight is essential. Despite these challenges, we are excited about advances in applying AI in mental health care, including the development of more sophisticated AI algorithms that show more human empathy and understanding and the integration of AI and other technologies for improved therapeutic experiences. *eBioMedicine* continues to be a publishing platform for translational studies that use AI for the diagnosis and treatment of people with mental disorders, as well as understanding the underlying mechanisms of these disorders.

